# Computer Tomograph (CT) imaging of mandibular anatomical substrate in animal model restored with nanostructured hydroxyapatite compounds

**Published:** 2015

**Authors:** R Ciuluvică, S Grădinaru, M Popescu, RM Piticescu, R Cergan

**Affiliations:** * Anatomy Department, “Carol Davila” University of Medicine and Pharmacy, Bucharest, Romania; ** Ophthalmology Department, “Carol Davila” University of Medicine and Pharmacy, Bucharest, Romania; *** Institute for Nonferous and Rare Materials, Bucharest, Romania

**Keywords:** hydroxyapatite, dental implant, bone reconstruction, nanostructured polymers

## Abstract

**Introduction:** This study was meant to test a new type of bone graft on an animal model. This material was a nanostructured hydroxyapatite.

**Materials and Methods:** the study was conducted according to Ethic Committee Regulation on animal model (Oryctolagus cuniculus – rabbit) between August and November 2014, at “Carol Davila” University of Medicine and Pharmacy, Bucharest. The animals were tested by using a CT at the level of the mandible before and after using the nanostructured hydroxyapatite.

**Results:** The animals were CT scanned at 1, 2 and respectively 3 months, noticing a growth of the mandibular bone density. After 3 months, a bone density equal with the density of the healthy bone was noticed.

**Conclusions:** The use of the bone graft could be a viable alternative to available materials. The advantage was that bone recovery had a density similar to the density of the healthy bone and the cost of production was low because it was made out of Calcium azotate and monobasic ammonium phosphate.

## Introduction

Bone graft materials are used in various orthopedic and maxillofacial surgery procedures for the treatment of bony defects caused by fractures, tumor resections or bone loss. In the last decades, different types of graft biomaterials with various origins have been developed, displaying specific advantages and disadvantages. In general, bone substitution materials can be categorized into natural bone grafts (autografts, allografts, xenografts, coralline) and synthetic bone grafts (alloplasts).

Hydroxyapatite is one of the materials frequently used in the reconstruction of dental bone defects.

This type of material is part of the category of biomaterials, which are classified as it follows [**1**-**5**]:

A) Autografs - autologous or autogenous bone grafting involves the use of the bone obtained from the same individual receiving the graft.

B) Allografts - Allograft is derived from humans. The difference is that the allograft is harvested from an individual other than the one receiving the graft [**6**-**8**].

C) Synthetic variants - Flexible hydrogel-hydroxyapatite (HA) composite which has a mineral.

D) Xenograft - Xenogratfs are bone grafts from a species other than human, such as bovine, and are used as a calcified matrix [**9**].

E) Alloplastic grafts - Alloplastic grafts may be made from hydroxyapatite, a naturally occurring mineral (main mineral component of bone) [**10**].

F) Ceramic-based bone graft substitutes (calcium sulfate, bioactive glass, and calcium phosphate) [**11**].

G) Polymer-based bone graft substitutes [**12**].

The materials must meet the following properties: osteoconduction, osteoinduction, and osteogenesis [**8**].

Depending on their use, bone grafts are available in different forms: hydroxyapatite granules of animal origin, spongious blocks of natural bone, elastic lamella of the cortical bone and formable, collagen paste from animal bone, etc. [**13**].

Calcium phosphates are the most abundant inorganic constituents of the living beings hard tissue, which provide bone and teeth with hardness, density and mechanical stability. Hydroxyapatite (Ca10(PO4)6(OH)2) is the main calcium phosphate in these tissues and the crystal unit cell comprises two entities.

The biomaterial used in our study is obtained from Calcium azotate and ammonium phosphate monobasic with a low risk of infectious disease, increased biocompatibility and low reactivity, polymers that were used for various medical applications, especially for dental surgery procedures [**14**-**16**].

Hydroxyapatite based nanostructured hybrids was synthesized by hydrothermal procedure at high pressure (>300 atm) and low temperatures (<200 °C), starting from Calcium azotate and ammonium phosphate monobasic and natural polymers such as type I collagen or alginate [**17**-**19**].

## Materials and methods

The purpose of this study was to reconstruct the mandibular osseous substrate with nanostructured hydroxyapatite from Calcium azotate and ammonium phosphate monobasic.

This study was rolled out from August to November 2014, on 20 male animal models - Oryctolagus cuniculus (rabbit), age 24 weeks following our Ethical Committee regulation regarding animal testing.

**Fig. 1:**
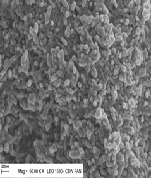
SEM characterization of hydroxyapatite nanopowder

Sterile nanostructured hydroxyapatite powder from Calcium azotate and ammonium phosphate monobasic as provided by Institute of Nonferrous and Rare Materials Bucharest after the chemical characterization and SEM characterization (**Fig. 1**).

In August, CT scans of rabbit mandible were performed and after the CT scan, the surgical step of this study, consisting in left incisive extraction and bone reconstruction by using nanostructured hydroxyapatite, was performed. CT scans assessed the bone density with SOMATOM – Siemens-software version syngoDENTAL CT2007E and 3D reconstruction made by 3D Recon software, HeadSpi/ Fine Sections program, which is used for routine investigations of the head, investigations of the base of the skull and brain, skull tumors, etc.

**Fig. 2:**
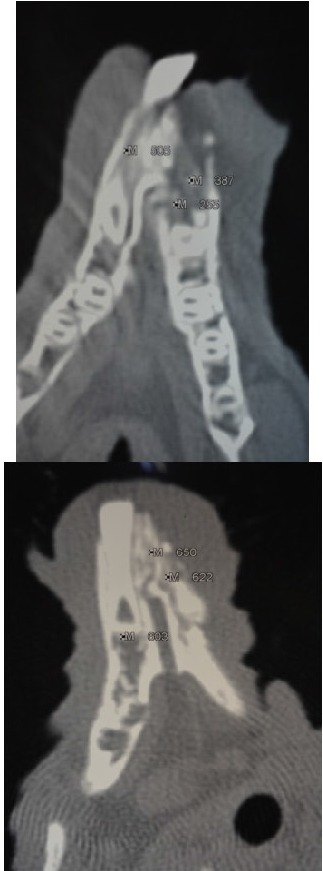
CT investigations at 1 month and 3 months after surgery showing central and peripheral bone density of mandible

## Results

Several CT image investigations were performed after the surgery by using the HeadSpi/ Fine sections 0.6-2.4 program.

The images were taken as it follows:

1) Topogram for choosing the segment of interest.

2) Initial aquisition of 2.4 in order to shorten the exposure and aquisition time for movements artifacts.

3) Reconstruction, HeadSecv 1.2, Headsecv. 0.6, and SSD (3D), frontal and sagital MPR range.

The following technical parameters were used (**Table 1**):

**Table 1:** Parameters used for CT scans (SOMATOM – Siemens-software version syngoDENTAL CT2007E)

kV	130
mAs	270
Slice col.	4x1 mm
Tomogram	256 mm
Slice width	2-4 mm
Feed/Rot	2.6 mm
Rot.time	0.75 s
Kernel	H 20s
Increm.	4-0,7mm
Direction	ca-cr
Scan range	40mm
CTDIw	71.5 m Gy

All the images were digitally reconstructed (**Table 2**), by using tri and bi-dimensional, frontal and sagittal reconstructions (MPR Range/ MPR Range 1).

**Table 2:** The technical parameters for reconstructions

Rec.	1.2-0.6mm
Kernel/bone	H 90 s ultra sharp
Kernel/orbit	H 60 s medium sharp

The purpose of these investigations was to make a comparison before and after surgery at the level of mandible (area of major interest in this study). The comparison was based on the parameters which resulted after the CT measurements on 150 relevant images for the study. A densitometry study was rolled out on these images. The animals were investigated at 1, 2 and 3 months. The bone density was measured in the mandibular region at the level of the insertion of the nanostructured hydroxyapatite.

The results are presented in the **Table 3**.

**Table 3:** Normal mandibular density preoperatively, and after 1, 2 and respectively 3 months after bone reconstruction

Normal mandibular bone density	Bone density 1 month after the reconstruction with nanostructured hydroxyapatite	Bone density 2 months after the reconstruction with nanostructured hydroxyapatite	Bone density 3 months after the reconstruction with nanostructured hydroxyapatite
500-550 UH	200-300 UH	300-400UH	500-600UH

## Discussion

Bone density increased in time after the surgery and 3 months after the surgery it reached a density equal with the density of the healthy bone. The obtained results were similar in all animals in the experiment.

Although there was a large variety of biomaterials for bone reconstruction, this nanostructured hydroxyapatite could provide an excellent support for dental implants.

## Conclusions

The use of nanotechnology in dentistry means the use of innovative materials, materials that are tested or are in the process of testing.

The conversion of nanostructured hydroxyapatite into a bone graft biomaterial presents multiple advantages not only from the medical point of view but also from the environmental and economic ones. The main advantage is the natural origin of the material to be created. Furthermore, nanostructured hydroxyapatite possesses excellent biocompatible and osteoconductive properties as well as mechanical and compressive strength similar with the human bone.

Following the results obtained, the nanostructured hydroxyapatite completes the range of bone graft biomaterials having a good biocompatibility and a low cost of production which could make it an accessible biomaterial.

**Acknowledgement**

This paper was supported by the Sectoral Operational Programme Human Resources Development (SOP HRD), financed from the European Social Fund and by the Romanian Government under the contract number POSDRU/159/1.5/S/132395”.
